# Hand proximity effects are fragile: a useful null result

**DOI:** 10.1186/s41235-018-0094-7

**Published:** 2018-03-28

**Authors:** Ronald Andringa, Walter R. Boot, Nelson A. Roque, Sadhana Ponnaluri

**Affiliations:** 1 Department of Psychology, Florida State University, 1107 W. Call Street, Tallahassee, FL 32306 USA; 2 Maclay School, Tallahassee, FL USA

## Abstract

Placing one’s hands near an object has been reported to enhance visual processing in a number of ways. We explored whether hand proximity confers an advantage when applied to complex visual search. In one experiment, participants indicated the presence or absence of a target item in a baggage x-ray image by pressing response boxes located at the edge of a tablet computer screen, requiring them to grip the display between their hands. Alternatively, they responded using a mouse held within their lap. Contrary to expectations, hand position did not influence search performance. In a second experiment, participants used their finger to trace along the x-ray image while searching. In addition to any effect of hand proximity it was predicted that this strategy would encourage a more systematic search strategy. Participants inspected bags longer using this strategy, but this did not translate into improved target detection. A third experiment attempted to replicate the near-hands advantage in a change detection paradigm featuring simple stimuli (Tseng and Bridgeman, Experimental Brain Research 209:257–269, 2011), and the same equipment and hand positions as Experiment 1, but was unable to do so. One possibility is that the grip posture associated with holding a tablet is not conducive to producing a near-hands advantage. A final experiment tested this hypothesis with a direct replication of Tseng and Bridgeman, in which participants responded to stimuli presented on a CRT monitor using keys attached to the side of the monitor. Still, no near-hands advantage was observed. Our results suggest that the near-hands advantage may be sensitive to small differences in procedure, a finding that has important implications for harnessing the near-hands advantage to produce better performance in applied contexts.

## Significance

Previous research has found that placing one’s hands around a display can enhance visual processing. The current study explored the generalizability of this effect, including to a complex search task. However, manipulations involving hand position failed to boost performance. Results have important implications for the generalizability of the near-hands advantage in applied contexts.

## Background

A growing literature suggests that when one’s hands are near an object, visual processing of that object is altered. For example, Reed, Grubb, and Steele (2006) explored the effect of hand position on the visual processing in peripersonal space (the space around one’s body). In a reaction time task, participants responded to targets appearing on the left or right side of a computer screen. They indicated the presence of the target by clicking a mouse button using one hand, and either held their other hand near or away from target locations on the screen. Facilitation (faster responses) was observed when a participant’s hand was held near where the target appeared, suggesting attentional prioritization of near-hand space. In subsequent experiments, Reed, Betz, Garza, and Roberts (2010) observed a response time benefit only when the target appeared near the participant’s palm rather than the back of their hand, consistent with a modulation of visual processing for objects within grasping space.

Changes in visual processing have also been observed for objects between the hands. Abrams, Davoli, Du, Knapp, and Paull (2008) had participants complete a variety of attention tasks by pushing buttons either on the side of the computer monitor (meaning the monitor was between their hands) or on a board placed in their lap. In a cuing paradigm, for example, Abrams et al. (2008) explored the effect of hand position on inhibition of return (IOR). IOR refers to an inhibitory effect observed when a location is cued and the target subsequently (after about 350 ms) appears in the same location (Wang & Klein, 2010). IOR effects are typically only observed after attention has been disengaged from a location, leading to the prediction that the increased scrutiny of, and delayed attentional engagement from, the cued location as a result of hand proximity would lead to a smaller IOR effect. This was exactly the pattern that was observed. Other experiments found an increase in reaction time slopes for a visual search task when hands were near the display, and an exaggeration of the attentional blink. Overall, Abrams et al. (2008) interpreted each of these findings as providing evidence that objects near the hands receive a more detailed analysis by the visual system, in part through a tendency to disengage attention more slowly.

Tseng and Bridgeman (2011) provided further evidence that stimuli near the hands receive enhanced processing. Methods were modelled after those of Abrams et al. (2008), except that participants performed a challenging change detection task. Participants were briefly presented with 8 or 12 colored squares. The display disappeared briefly and then reappeared, with or without a change in color of one square. Sensitivity to change was significantly greater when participants’ hands were near the monitor. Further, this facilitation appeared to be present regardless of whether the change was near the hands or far from the hands, as long as the change occurred between them.

Hand position has also been found to reduce distraction. Davoli and Brockmole (2012) had participants complete a task in which they identified a letter at the center of the screen surrounded by two large flanking letters. Responses are typically slowed when flanking letters suggest a different response compared to the center letter (flanker effect). The critical manipulation involved whether or not the center letter appeared between participants’ cupped hands. When it did, participants were able to completely ignore the flanking items, in contrast to a large flanker effect when their hands were away from the display. Artificial hand-shaped barriers did not have the same effect. These results suggest that, in addition to enhancing visual processing, holding an object reduces distraction from items not within one’s hands.

Interestingly, semantic processing of words presented between the hands appears to be impaired, consistent with a decrease in holistic analysis and a greater focus on visual detail. Davoli, Du, Montana, Garverick, and Abrams (2010) presented sentences between participants’ hands and asked them to judge whether or not they made sense. Detection of nonsense sentences between the hands was impaired. In a follow-up experiment, participants completed a Stroop task (naming the font color of a word while ignoring the word’s meaning). Stroop interference was decreased when the word was presented between participants’ hands. The authors interpreted this as a decrease in semantic processing and an increase is spatial processing.

The neural mechanisms responsible for changes in visual processing based on hand proximity are not fully understood (Brockmole, Davoli, Abrams, & Witt, 2013). Many explanations implicate multimodal neurons. Unlike unimodal neurons, which only respond to one stimulus modality (e.g., taste, sound, touch, sight, smell), multimodal neurons receive and integrate information from two or more modalities (Stein & Stanford, 2008). For example, bimodal visuotactile neurons respond to both visual and tactile stimulation (Bresciani, Dammeier, & Ernst, 2006; Graziano & Gross, 1998). Visuotactile neurons in premotor and parietal cortex in particular respond to objects near the hands and have hand-centric receptive fields (Tseng, Bridgeman, & Juan, 2012). These neurons can even exhibit a graded response, exhibiting greater activation as the distance between the hand and an object decreases. It is possible that such bimodal neurons provide additional neural signals with which to detect and process items close to one’s hands. The presence of these neurons specifically in parietal cortex, which plays a large role in spatial attention, further links hand position to potential changes in visual processing and attentional disengagement.

The literature reviewed here suggests that (1) objects within the hands receive a more careful analysis compared to objects outside of the hands, (2) attention is biased toward space near the hands, (3) distraction can be prevented for objects held within one’s hands, and (4) processing can become more detail-oriented for objects within one’s hands, shifting from a holistic/semantic processing mode to a spatial one. Given these processing advantages, it is possible that the near-hands effect might be harnessed to improve the performance of real-world tasks. However, it is important to note that all of these previous studies have used relatively simple and abstract tasks and stimuli and it is not clear how well these effects might scale up to more demanding naturalistic tasks, and whether these effects are powerful enough to improve performance when stimuli are significantly more complex. Here, we explore these questions in the context of a difficult real-world search task, that of x-ray baggage screening. To preview our results, we observed no effect of hand position in four different experiments that varied stimulus complexity and grip posture, suggesting that the near-hands advantage is fragile and may be influenced by many factors. This has important implications for harnessing the near-hands advantage to produce better performance of complex tasks.

## Experiment 1

### Methods

#### Participants

Sixty-eight undergraduate students at Florida State University with self-reported normal color vision participated for course credit (*M* age = 18, *SD* = 1.03; 11 males).

#### Materials

All stimuli were presented on a 12-in. Microsoft Surface Pro 3 tablet PC (1280 × 768 resolution). Response latency and accuracy data were collected using either a mouse or touch screen responses.

#### Stimuli

**Fig. 1:**
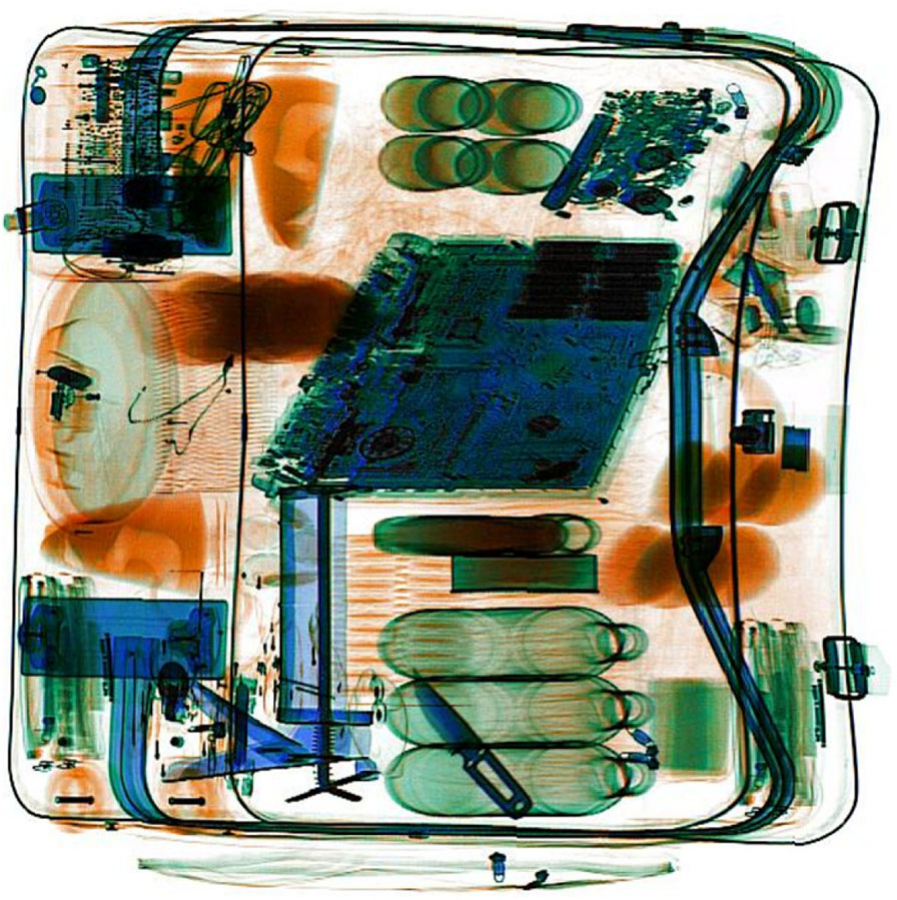
Example of a target-present image. The knife is in the lower portion of the image, at center

Stimulus sets were generated using images created for a previous study (McCarley, 2009). Actual x-ray images of bags, clutter (non-target) items, and target items (knives) were digitally combined to create realistically cluttered bags (see McCarley, 2009 for more details). This original set included 900 x-ray images of baggage. Smaller bags were excluded from the present study to enhance the perception that the full extent of the bag was between participants’ hands. This process identified 229 images of baggage with a target (knife) present and 292 bags containing no target (Fig. 1). In a pilot study, five participants searched these images and responded whether a target was present or absent. We used accuracy data from these participants to create two sets of 120 images of approximately equal difficulty that were counterbalanced across conditions in a within-participant design. We will refer to these image sets as Set A and Set B. Each set had 60 target-present images and 60 target-absent images.

#### Baggage screening paradigm

**Fig. 2:**
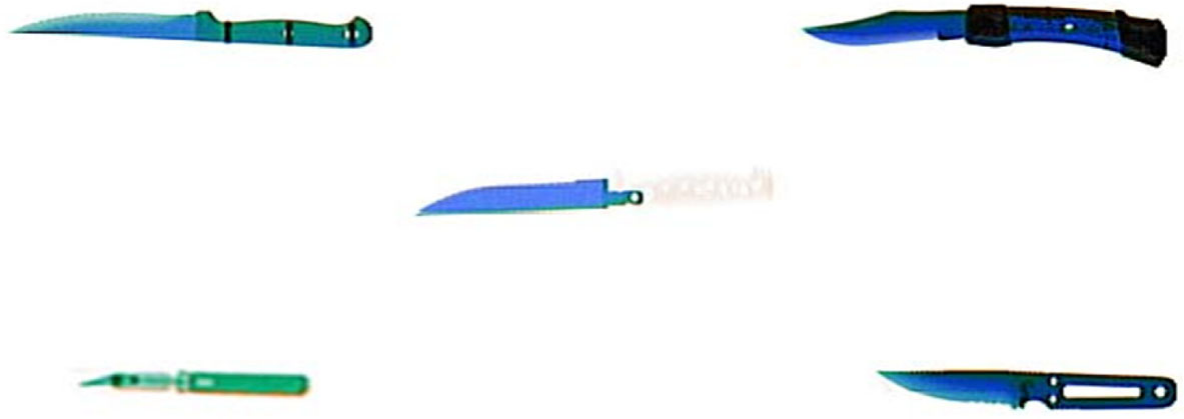
Image of the five knives that could appear in bags. Before each block of trials, participants were shown this image to familiarize them with potential targets

**Fig. 3:**
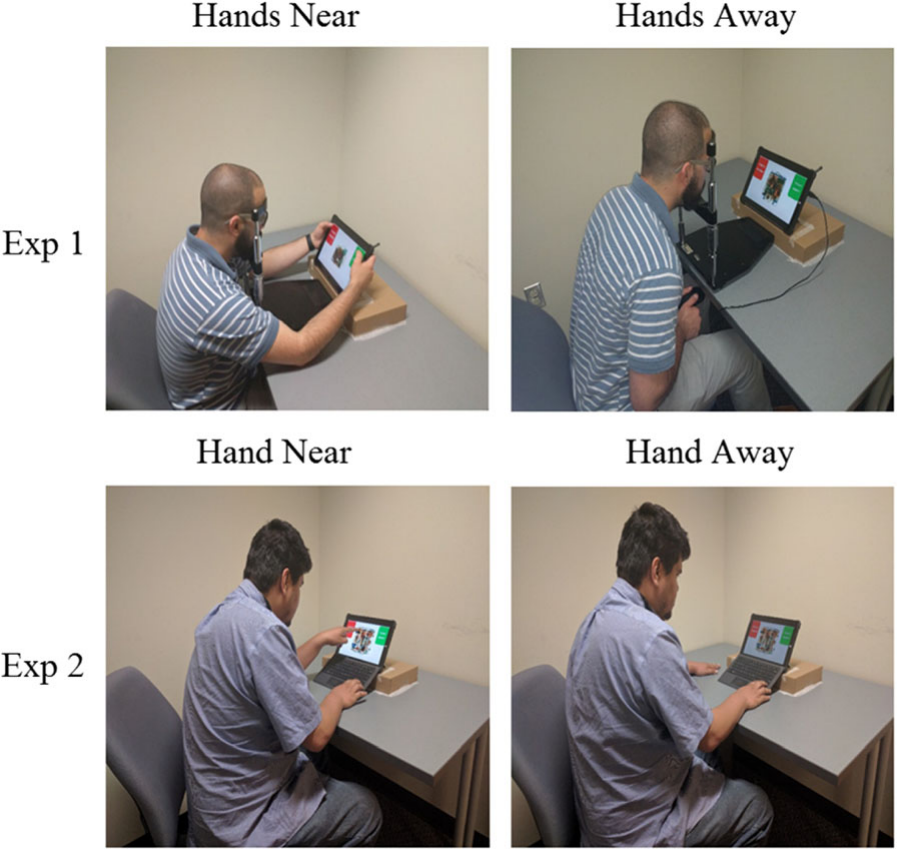
Depiction of the experimental setup and hand positions for Experiments 1 and 2

The baggage paradigm was created using OpenSesame (Mathôt, Schreij, & Theeuwes, 2012). Participants were asked to report whether a target was present or absent by making one of two responses. Participants were familiarized with all five target knives before each block of trials (Fig. 2). In one version of the task, participants made threat-present and -absent responses using response boxes near the edges of the screen of the tablet implemented through the touchscreen interface. The position of these response boxes required that the tablet be held between the hands of the participant (Hands Near condition, Fig. 3). In another version, images of these response boxes remained on the screen, but participants responded by pressing the left or right mouse button of a mouse held in their lap (Hands Away condition, Fig. 3). No feedback was provided regarding response accuracy.

#### Procedure

Protocols for all experiments were approved by Florida State University’s Human Subjects Committee (HSC No. 2016.17423). After giving consent, participants completed the x-ray baggage paradigm twice. Hand position and image set order were counterbalanced. In each condition, participants were told to imagine that they were a worker at an airport-security station, and their job was to search for hidden knives in images of luggage. If participants saw a bag containing a knife they were instructed to respond Threat Present. If participants did not see a knife they were instructed to respond Threat Absent. In the Hands Near condition, participants responded by pressing the Threat Present box on the screen with their left thumb and Threat Absent box on the screen with the right thumb. In the Hands Away condition, participants responded by pressing the left mouse button for a Threat Present response with the left thumb and pressed the right mouse button for a Threat Absent response with the right thumb. The experimenter read instructions directly from the screen, word for word, emphasizing the idea that participants should respond accurately, but not take any more time than necessary. Each block of 120 trials was preceded by 10 practice trials (5 present, 5 absent).

### Results

#### Trial exclusion

Response time analyses only included trials on which participants made a correct response.

#### Analysis approach

Means and standard deviations for all conditions are presented in Table 1 for Experiments 1 and 2. To supplement null hypothesis tests reported below, Table 1 also reports Bayes factors (B_10_) associated with *t* tests contrasting Hands Near and Hands Away conditions (Rouder, Speckman, Sun, Morey, & Iverson, 2009). Bayes factors indicate how strongly the data favor the null or alternative hypothesis and are reported as likelihood ratios with the evidence for the null model in the denominator; a B_10_ value less than 1.0 provides evidence in favor of a null effect and a B_10_ value of greater than 1.0 provides evidence for a non-null effect. Terms used to discuss the evidential impact of reported Bayes factors are borrowed from Wetzels et al. (2011), and are as follows: anecdotal or worth no more than a bare mention (1/3 < B_10_ < 3), substantial (1/10 < B_10_ ≤ 1/3 or 3 ≤ B_10_ < 10), strong (1/30 < B_10_ ≤ 1/10 or 10 < B_10_ < 30), very strong (1/100 < B_10_ ≤ 1/30 or 30 ≤ B_10_ < 100), and decisive (B_10_ < 1/100 or B_10_ > 100).

**Table 1:** Means, standard deviations, and Bayes factors (B_10_) for each condition in Experiments 1 and 2

Measure	Experiment 1			Experiment 2		
	Hands Near	Hands Away	B_10_	Hand Near	Hand Away	B_10_
RT (ms), target present	3751 (1913)	3473 (1773)	1/2.46	4515 (1573)	3276 (1268)	2812/1
RT (ms), target absent	7157 (3726)	6901 (3516)	1/5.92	8831 (4470)	6240 (3247)	310/1
Accuracy (prop), target present	0.51 (0.13)	0.50 (0.11)	1/7.16	0.53 (0.13)	0.53 (0.12)	1/4.62
Accuracy (prop), target absent	0.88 (0.13)	0.89 (0.12)	1/6.32	0.90 (0.08)	0.88 (0.09)	1/3.46
Sensitivity (*A*)	0.80 (0.08)	0.79 (0.09)	1/7.50	0.81 (0.06)	0.80 (0.05)	1/3.60
Bias (log (*b*))	0.72 (0.45)	0.73 (0.38)	1/7.38	0.69 (0.31)	0.67 (0.36)	1/4.96

#### Accuracy

Accuracy and threat sensitivity data were of primary interest. Accuracy data were entered into an ANOVA with *Target Presence* (Present vs. Absent) and *Hand Position* (Hands Near vs. Hands Away) as within-participant factors. A main effect of *Target Presence* was found, *F*(1,67) = 256.98, *P* < 0.001, indicating participants were more accurate on trials in which threats were absent. Contrary to predictions, *Hand Position* had no effect on accuracy, *F*(1,67) = 0.003, *P* = 0.96, and there was no interaction between *Hand Position* and *Target Presence*, *F*(1,67) = 0.27, *P* = 0.61. According to calculated Bayes factors, the target present and absent conditions provided substantial evidence in favor of the null hypothesis.

#### Sensitivity

We considered target sensitivity (*A*) as the primary measure of performance. *A* is a corrected measure of the sensitivity measure *A’* (Zhang and Mueller, 2005). It is an alternative to *d’* and is useful in cases in which hit rates are sometimes 100% or false alarm rates are zero. It was predicted that, if hand position encourages more thorough and detailed visual processing, sensitivity would be higher in the Hands Near condition. Sensitivity data were entered into an ANOVA with *Hand Position* (Hands Near vs. Hands Away) as a within-participant factor. Contrary to predictions, there was no effect of *Hand Position*, *F*(1,67) = 0.004, *P* = 0.95. According to the calculated Bayes factor, data provided substantial evidence in favor of the null hypothesis.

#### Bias

In addition to sensitivity, we also explored whether hand position might influence bias. Natural log (*b*) served as a measure of bias, and is a symmetric bias measure associated with *A* (Zhang and Mueller, 2005). Bias data were entered into an ANOVA with *Hand Position* (Hands Near vs. Hands Away) as a within-participants factor. There was no effect of *Hand Position* on bias, *F*(1,67) = 0.04, *P* = 0.85. According to the calculated Bayes factor, data provided substantial evidence in favor of the null hypothesis.

#### Response time

Given that mouse and touchscreen inputs required different motor actions that may be more or less difficult to perform, response time data in Experiment 1 (and later Experiment 3) are not very informative regarding visual processing (i.e., pressing buttons on a mouse may simply be easier or harder than using a touchscreen). However, participants’ response times were entered into an ANOVA with *Target Presence* (Present vs. Absent) and *Hand Position* (Hands Near vs. Hands Away) as within-participant factors. A main effect of *Target Presence* was found, *F*(1,67) = 138.05, *P* < 0.001. Participants were slower to respond on trials in which threat items were absent compared to present, just as expected in a self-terminating search task. However, *Hand Position* had no effect on response times, *F*(1,67) = 1.05, *P* = 0.31, nor did it interact with *Target Presence*, *F*(1,67) = 0.009, *P* = 0.93. According to calculated Bayes factors, the target-absent condition provided substantial evidence in favor of the null hypothesis, while the target-present condition provided anecdotal evidence in favor of the null.

#### Summary

We predicted that participants in the Hands Near condition would exhibit enhanced visual processing that would produce increased accuracy and greater sensitivity to target items. Contrary to predictions, search performance did not differ as a function of participants’ hand position. Overall, data were consistent with the null hypothesis.

## Experiment 2

One potential reason for a lack of a near-hands effect might be that the search targets were too far from the participants’ hands. At least some effects require the hand to be near the target for benefits to be observed (e.g., Reed et al., 2006). A second experiment was conducted with the purpose of decreasing the distance between participants’ hands and the search target. We asked participants to perform the task using a “Finger Sweep” strategy in which they traced the finger of one hand systematically along the image during search to ensure the proximity of their hand to the search target on each trial. We anticipated that this strategy might benefit search for two reasons. In addition to any potential benefit in terms of visual processing resulting from hand proximity, we predicted that this strategy would also encourage a more systematic search, shifting participants toward a strategy of more careful scanning in which more potential target locations would be inspected (McCarley, 2009).

### Methods

#### Participants

Thirty-two undergraduate students with self-reported normal color vision participated in this study for course credit (*M* age = 19, *SD* = 1.45; 9 males).

#### Materials

Same as Experiment 1.

#### Baggage screening paradigm

The paradigm was identical to the one used in Experiment 1 except that the response method and hand positions differed. Participants in both conditions reported whether a target was present or absent using one of two keys on the keyboard using their right hand. In one condition a “Finger Sweep” strategy was introduced in which participants were encouraged to trace their left index finger along the image, left to right, top to bottom (Hand Near condition, Fig. 3). In another condition participants placed their left hand on the table away from the display (Hand Away condition, Fig. 3).

#### Procedure

Hand position and image set order were counterbalanced in a within-participant design. In the Hand Near condition, participants were told to use a specific strategy. This strategy involved putting the left index finger on the screen. Starting at the upper left-hand corner of the bag, participants were to sweep their finger from left to right across the bag making a horizontal line, and would then start again below the previously traced line. Participants were instructed to search the area of the bag where their finger was located and to move their attention as their finger moved. They were told the aim of this strategy was to help make sure that all locations in which a target could be located received attention. If a target was found, participants were told to stop scanning the bag with their finger and make a response. A video of the “Finger Sweep” strategy was displayed for the participant and they were told to emulate the video for each image. If a target was present participants responded by pressing the left arrow key and if a target was absent participants responded by pressing the right arrow key (using their right hand).

In the Hand Away condition participants were told to search for a knife while the left hand was held flat against the table to the left of the keyboard. For each condition, experimenters read instructions directly from the screen, word for word, emphasizing the idea that participants should respond accurately, but not to take any more time than necessary. Each block was preceded by 10 practice trials.

### Results

#### Trial exclusion

Only response times from accurate trials were considered in analyses of response time.

#### Accuracy

Accuracy data were entered into an ANOVA with *Target Presence* (Present or Absent) and *Hand Position* (Hand Near vs. Hand Away) as within-participant factors. A main effect of *Target Presence* was found, *F*(1,31) = 143.96, *P* < 0.001, indicating that participants were more accurate on trials in which threats were absent compared to trials on which threats were present. However, contrary to prediction, *Hand Position* had no effect on accuracy, *F*(1,31) = 1.28, *P* = 0.27, and there was no interaction between *Hand Position* and *Target Presence*, *F*(1,31) = 0.068, *P* = 0.80. According to calculated Bayes factors, the target present and absent conditions provided substantial evidence in favor of the null hypothesis.

#### Sensitivity

Sensitivity data (*A*) were entered into an ANOVA with *Hand Position* (Hand Near vs. Hand Away) as a within-participant factor. Contrary to a near-hand advantage, there was no effect of *Hand Position* on sensitivity, *F*(1,31) = 0.84, *P* = 0.37. According to the calculated Bayes factor, data provided substantial evidence in favor of the null hypothesis.

#### Bias

Bias data (log (*b*)) were entered into an ANOVA with *Hand Position* (Hand Near vs. Hand Away) as a within-participant factor. There was no effect of *Hand Position* on bias, *F*(1,31) = 0.14, *P* = 0.71. According to the calculated Bayes factor, data provided substantial evidence in favor of the null hypothesis.

#### Response time

Response times were entered into an ANOVA with *Target Presence* (Present vs. Absent) and *Hand Position* (Hand Near vs. Hand Away) as within-participant factors. This analysis indicated a significant effect of *Target Presence*, *F*(1,31) = 67.52, *P* < 0.001, *Hand Position*, *F*(1,31) = 27.14, *P* < 0.001, and a significant interaction between *Target Presence* and *Hand Position*, *F*(1,31) = 8.36, *P* < 0.01. The finger sweep condition prolonged inspection times, and this was especially true for target absent trials (increase of 1238 ms for target present trials, 2590 ms for target absent trials). According to calculated Bayes factors, the target present and absent conditions provided decisive evidence in favor of an effect of hand position.

#### Summary

Participants were asked to sweep the screen with a finger to make sure all possible locations were searched. This made them substantially slower, especially on target absent trials, but contrary to expectations, this extra time viewing each image did not improve sensitivity.

## Experiment 3

It is unclear whether the lack of a near-hands advantage might be due to the complexity of the search task compared to previous studies, or due to the specific grip postures and response requirements of our experiment compared to previous studies in which participants pushed buttons attached to the side of a computer monitor. An additional experiment was conducted to help distinguish between these possibilities. Participants performed a change detection task similar to the one reported by Tseng and Bridgeman (2011), but on a tablet with their hands either around the tablet or in their laps (postures and responses identical to Experiment 1 of the current manuscript). Participants viewed a number of colored squares for a short period of time. These squares disappeared, and then reappeared again a short time later. The task of the participant was to judge whether or not one of the squares changed color when the display reappeared.

### Methods

#### Participants

Fifty-six undergraduate students at Florida State University with self-reported normal or corrected-to-normal vision participated for course credit. Participants were also screened for color blindness using self-report and a subset of the plates from the Ishihara color blindness test: plate one (numeral twelve), plate nine (numeral seventy-four), and plate eleven (numeral six). In total, five participants were excluded from analysis (two due to color deficiency, three due to missing data). Fifty-one participants were included in most reported analysis (*M* age = 20.06, *SD* = 2.30; 23 males). In one condition (Set Size 12 – Hands Away), one additional participant had more false alarms than hits, making it impossible to calculate bias. This participant was excluded from sensitivity and bias analyses.

#### Materials

Same as previous experiments.

#### Stimuli

Stimuli for the visual short-term memory task were displayed within a 14.4 × 14.4 degree region at the center of the tablet screen. These stimuli consisted of colored squares (0.67 degrees), separated by a minimum edge-to-edge distance of 0.54 degrees, on a gray background. Squares were randomly assigned the colors green, magenta, red, yellow, blue, white, and black. The display was organized in an invisible 6 × 6 grid with a total of 36 possible locations in which squares could appear. Targets (squares that changed color) could only appear in each location once during a block, producing a total of 36 ‘change’ and 36 ‘no-change’ trials. When a square changed color, the new color was randomly selected from a list of possible colors excluding the current color. Set sizes (number of squares on the screen) of 8 and 12 were used. Trials began with an 800 ms fixation, followed by a 200 ms blank display. Then the memory set appeared for 250 ms, followed by a 900 ms delay period, and finally the test display for 2200 ms. Participants made their response during the test display and trials timed out if they took longer than 2200 ms to respond. About 1.4% of all responses were not made within this response window, with these responses being distributed equally across conditions.

#### Procedure

Participants sat approximately 42.5 cm from the screen, with distance being controlled by a chin rest, and made responses similar to Experiment 1 (Fig. 3, top). The “Threat Present” button was relabeled “Change”, and the “Threat Absent” button was relabeled “No Change.” The colors of these buttons were changed to black font on a gray background to prevent interference with the color memory task. Participants completed four blocks of trials that varied in set size (number of items in the display) and hand position: Set Size 8 – Hands Near, Set Size 12 – Hands Near, Set Size 8 – Hands Away, Set Size 12 – Hands Away. Each block contained 72 trials (50% change trials). All blocks were completed in counterbalanced order. A practice block of 20 trials preceded the first block, with this practice block containing the same number of items as the initial block participants completed.

### Results

#### Trial exclusion

For response time analyses, only accurate trials were considered. The small number of trials on which a response was not made were not considered in reported analyses.

#### Accuracy

All data are depicted in Table 2, along with calculated Bayes factors. Accuracy data were entered into an ANOVA with *Hand Position* (Near vs. Away), *Set Size* (8 vs. 12), and *Trial Type* (Change vs. No Change) as within-participant factors. As expected, accuracy was worse for larger set sizes, *F*(1,50) = 166.32, *P* < 0.001. Accuracy was also worse for change trials compared to no-change trials, *F*(1,50) = 35.30, *P* < 0.001. Set size and trial type interacted such that accuracy was especially poor for larger set size change trials, *F*(1,50) = 34.91, *P <* 0.001. There was no effect of *Hand Position*, *F*(1,50) = 0.37, *P =* 0.55, and hand position did not interact with *Set Size*, *F*(1,50) = 0.32, *P =* 0.57, or *Trial Type*, *F*(1,50) = 3.09, *P =* 0.09. Unexpectedly, instead of a clear near-hands advantage, there was a significant three-way interaction between *Hand Position*, *Set Size*, and *Trial Type*, *F*(1,50) = 8.16, *P <* 0.01. This interaction appeared to be driven by higher accuracy when the hands were near the display in the large set size no-change condition compared to when the hands were away from the display in the same condition (*M* = 0.79, *SD* = 0.13 vs. *M* = 0.75, *SD* = 0.13; *t*(50) = 3.20, *P* < 0.01). No other conditions differed when hands were near compared to away (all *P* values > 0.12). According to calculated Bayes factors, in general, there was substantial evidence for the null in all conditions except for when the set size was large and no change occurred. In this case alone there was substantial evidence for a near-hands advantage. Overall, these results provide little evidence for a robust near-hands advantage. Next, we turn to measures of sensitivity and bias.

**Table 2:** Means, standard deviations, and Bayes factors (B_10_) for each condition in Experiments 3

Measure	Set Size 8			Set Size 12		
	Hands Near	Hands Away	B_10_	Hand Near	Hand Away	B_10_
RT (ms), change	1074 (146)	936 (155)	68,126,027/1	1113 (167)	919 (184)	85,180,554,982/1
RT (ms), no-change	1062 (172)	961 (195)	84,511/1	1102 (193)	950 (213)	2,222,248/1
Accuracy (prop), change	0.72 (0.15)	0.72 (0.17)	1/6.36	0.53 (0.16)	0.56 (0.16)	1/1.61
Accuracy (prop), no-change	0.82 (0.11)	0.82 (0.13)	1/6.33	0.79 (0.13)	0.75 (0.13)	8.43/1
Sensitivity (*A*)	0.84 (0.08)	0.84 (0.10)	1/6.50	0.74 (0.10)	0.73 (0.08)	1/4.54
Bias (log (*b*))	0.20 (0.36)	0.22 (0.41)	1/6.14	0.41 (0.42)	0.27 (0.36)	3.44/1

#### Sensitivity

Sensitivity data (*A*) were entered into an ANOVA with *Hand Position* (Near vs. Away) and *Set Size* (8 vs. 12) as within-participant factors. Sensitivity was the primary measure of interest reported by Tseng and Bridgeman (2011). A main effect of *Set Size* was observed, with greater sensitivity for change when the set size was smaller, *F*(1,49) = 132.50, *P* < 0.001. Contrary to expectations, no effect was observed for *Hand Position*, *F*(1,49) = 0.62, *P* = 0.43. *Hand Position* and *Set Size* did not interact *F*(1,49) = 0.40, *P* = 0.53. According to calculated Bayes factors, there was substantial evidence for the null in both the small and large set size conditions.

#### Bias

Bias data (log (*b*)) were entered into an ANOVA with *Hand Position* (Near vs. Away) and *Set Size* (8 vs. 12) as within-participant factors. This analysis revealed a main effect of *Set Size*, *F*(1,49) = 9.08, *P* < 0.01. Participants were more conservative (less likely to say “change”) when the set size was larger. There was no effect of *Hand Position*, *F*(1,49) = 2.54, *P* = 0.12. However, consistent with the previously reported accuracy analysis, there was an interaction between *Hand Position* and *Set Size*, *F*(1,49) = 5.41, *P* < 0.05. In the small set size conditions, hand position made no difference, *t*(49) = −0.35, *P* = 0.73. However, in the large set size condition, participants were more conservative when their hands were around the display, *t*(49) = 2.64, *P* < 0.05. This is consistent with the accuracy analysis indicating significantly fewer false alarms (higher no-change accuracy) in the large set size condition when participants had their hands around the display. According to Bayes factors, there was substantial evidence for the null in the small set size condition, but substantial evidence in favor of a hands effect in the large set size condition. In general, results were inconsistent with the large improvements in change sensitivity observed by Tseng and Bridgeman (2011).

#### Response time

Although response time data were not of primary interest to this paradigm since changes may reflect differences in difficulty between using a mouse compared to a touchscreen to respond, they were entered into an ANOVA with *Hand Position* (Near vs. Away), *Set Size* (8 vs. 12), and *Trial Type* (Change vs. No Change) as within-participant factors. No effect was observed of *Set Size*, *F*(1,50) = 0.45, *P* = 0.51, or *Trial Type*, *F*(1,50) = 0.28, *P* = 0.60. However, there was a robust effect of *Hand Position*, *F*(1,50) = 123.60, *P* < 0.001. In general, participants were 146 ms slower when their hands were near the display compared to away. This was similar to the general pattern observed in Experiment 1 using the same hand positions and responses, though in Experiment 1 this difference was not significant. There were also significant interactions between *Hand Position* and *Set Size*, *F*(1,50) = 7.25, *P* < 0.05, and *Hand Position* and *Trial Type*, *F*(1,50) = 10.61, *P* < 0.01. There was, in general, a greater increase in response time for the hands-near condition when the set size was larger (Set 8: 120 ms difference vs. Set 12: 173 ms difference). Further, while no-change trials were faster than change trials when the hands were near (11 ms), change trials were faster than no-change trials when the hands were away (28 ms). The three-way interaction between *Hand Position*, *Set Size*, and *Trial Type* was not significant, *F*(1,50) = 0.049, *P* = 0.83. According to calculated Bayes factors, there was decisive evidence of a hands effect in all conditions. Participants were slower when their hands were near the display.

#### Summary

The most striking finding was a slowing when the hands were near the stimuli compared to away, with weaker evidence that this effect interacted with other factors. While this might be interpreted as an effect of hand proximity, it likely reflects greater physical difficulty participants had interacting with touchscreen buttons compared to physical buttons held within their hands. Some evidence was obtained for more conservative responses when participants had their hands near the display and the set size was large. However, in general, the pattern of results was inconsistent with what was reported by Tseng and Bridgeman (2011). This provides evidence that the specific device, grip postures, and responses used in our experiments may be responsible for the lack of a near-hands advantage. It is interesting that previous experiments have tended to ask participants to use fairly novel grip postures (it is not very common for people to push buttons attached to the side of a CRT monitor), while the current Experiments 1–3 used a more familiar grip; it is common for individuals to manipulate a touchscreen (tablet or smartphone) using their thumbs (e.g., when scrolling through emails or flipping the pages of an eBook). Thomas (2017) has shown that previous experience can shape the size and nature of the near-hands advantage, and it is possible that participants’ previous tablet and smartphone experience may have also interfered with our ability to observe a near-hands advantage.

## Experiment 4

It is unclear whether the lack of a near-hands advantage might be due to the specific grip postures and response requirements of our experiment compared to previous studies in which participants pushed buttons attached to the side of a computer monitor. To examine this possibility, another experiment was conducted in which participants were asked to complete the same task as Experiment 3, but responded to stimuli presented on a CRT monitor using keys attached to the side of the display, similar to procedure of Tseng and Bridgeman (2011).

### Methods

#### Participants

Fifty-nine undergraduate students at Florida State University with self-reported normal or corrected-to-normal visual acuity participated for course credit. Participants were also screened for color blindness through self-report and a subset of plates from the Ishihara color blindness test: plate one (numeral twelve), plate nine (numeral seventy-four), and plate eleven (numeral six). Eleven participants were initially excluded from analysis (five due to color deficiency, six due to missing data). However, in one or more conditions, eight participants had more false alarms than hits, making it impossible to calculate bias. As in previous experiments, these eight participants were excluded from sensitivity and bias analyses. However, because these cases may also represent instances of key confusion (participants reversing the “change” and “no-change” keys), these participants were also excluded from accuracy and response time analyses. This resulted in a final sample size of 40 participants (*M* age = 18.48, *SD* = 0.72; 11 males). The pattern of significance and Bayes factors was unaffected when these participants were included.

#### Materials

Stimuli were presented with a Dell Optiplex computer on a 21 in. NEC AccuSync 120 - CRT monitor (1280 × 768 resolution). Response latency and accuracy data were collected using a USB numeric keypad.

#### Stimuli

Same as Experiment 3. Stimuli size was adjusted so that visual angle was equated with Experiment 3.

#### Procedure

**Fig. 4:**
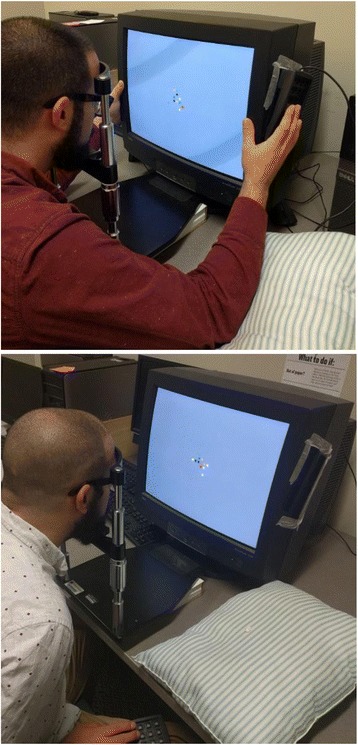
Depiction of the experimental setup and hand positions for Experiments 4

Participants sat approximately 28 cm from the monitor, with distance controlled by a chin rest. In the Hands Near conditions, participants held their hands around the monitor. Participants pressed the 1 key on the keypad attached to the side of the monitor for “Change,” and the 2 key for “No Change.” In the Hands Away condition participants had their hands in their lap (Fig. 4). Velcro was used to secure the keypad to the side of the monitor so it could easily be moved from the monitor to the participant’s lap and back again when necessary.

### Results

#### Trial exclusion

For response time analyses, only accurate trials were considered. The small number of trials on which a response was not made were not considered in reported analyses.

#### Accuracy

Data are presented in Table 3, along with calculated Bayes factors. Accuracy data were entered into an ANOVA with *Hand Position* (Near vs. Away), *Set Size* (8 vs. 12), and *Trial Type* (Change vs. No Change) as within-participant factors. As expected, accuracy was worse for larger set sizes, *F*(1,39) = 104.56, *P* < 0.001. Accuracy was also worse for change trials compared to no-change trials, *F*(1,39) = 39.37, *P* < 0.001. Set size and trial type interacted such that accuracy was especially poor for larger set size change trials, *F*(1,39) = 27.85, *P <* 0.001. There was no effect of *Hand Position*, *F*(1,39) = 0.50, *P =* 0.49, and hand position did not interact with *Set Size*, *F*(1,39) < 0.01, *P* = 0.99, or *Trial Type*, *F*(1,39) = 0.10, *P* = 0.76. The interaction between *Hand Position*, *Set Size*, and *Trial Type* was not significant, *F*(1,39) = 0.27, *P =* 0.61. According to calculated Bayes factors, there was substantial evidence in favor of the null with respect to hand position. Next, we turn to measures of sensitivity and bias.

**Table 3:** Means, standard deviations, and Bayes factors (B_10_) for each condition in Experiments 4

Measure	Set Size 8			Set Size 12		
	Hands Near	Hands Away	B_10_	Hand Near	Hand Away	B_10_
RT (ms), change	843 (151)	847 (144)	1/5.74	877 (179)	881 (172)	1/5.75
RT (ms), no-change	839 (162)	843 (176)	1/5.80	874 (248)	846 (226)	1/3.08
Accuracy (prop), change	0.72 (0.15)	0.71 (0.15)	1/5.71	0.53 (0.13)	0.52 (0.14)	1/4.81
Accuracy (prop), no-change	0.84 (0.11)	0.83 (0.12)	1/5.46	0.79 (0.14)	0.79 (0.15)	1/5.81
Sensitivity (*A*)	0.84 (0.09)	0.84 (0.07)	1/5.84	0.74 (0.08)	0.74 (0.07)	1/5.86
Bias (log (*b*))	0.24 (0.38)	0.24 (0.43)	1/5.86	0.44 (0.41)	0.46 (0.44)	1/5.59

#### Sensitivity

Sensitivity data (*A*) were entered into an ANOVA with *Hand Position* (Near vs. Away) and *Set Size* (8 vs. 12) as within-participant factors. Sensitivity was the primary measure of interest reported by Tseng and Bridgeman (2011). A main effect of *Set Size* was observed, with greater sensitivity for change when the set size was smaller, *F*(1,39) = 65.76, *P* < 0.001. Contrary to expectations, no effect was observed for *Hand Position*, *F*(1,39) = 0.003, *P* = 0.95. *Hand Position* and *Set Size* did not interact *F*(1,39) = 0.001, *P* = 0.97. According to calculated Bayes factors, there was substantial evidence in favor of the null with respect to hand position.

#### Bias

Bias data (log (*b*)) were entered into an ANOVA with *Hand Position* (Near vs. Away) and *Set Size* (8 vs. 12) as within-participant factors. This analysis revealed a main effect of *Set Size*, *F*(1,39) = 15.14, *P* < 0.001. Participants were more conservative (less likely to say “change”) when the set size was larger. There was no effect of *Hand Position*, *F*(1,39) = 0.04, *P* = 0.83. Further, there was no interaction between *Hand Position* and *Set Size*, *F*(1,39) = 0.04, *P* = 0.83. According to calculated Bayes factors, there was substantial evidence for the null with respect to the effect of hand position.

#### Response time

Response time data were entered into an ANOVA with *Hand Position* (Near vs. Away), *Set Size* (8 vs. 12), and *Trial Type* (Change vs. No Change) as within-participant factors. No effect was observed of *Set Size*, *F*(1,39) = 1.32, *P* = 0.26, *Trial Type*, *F*(1,39) = 0.55, *P* = 0.47, or *Hand Position*, *F*(1,39) = 0.11, *P* = 0.75. There were no significant interactions between *Hand Position* and *Set Size*, *F*(1,39) = 0.34, *P* = 0.57, or *Hand Position* and *Trial Type*, *F*(1,39) = 2.41, *P* = 0.13. The three-way interaction between *Hand Position*, *Set Size*, and *Trial Type* was not significant, *F*(1,39) = 2.57, *P* = 0.12. According to calculated Bayes factors, there was substantial evidence for the null in all conditions with respect to hand position.

#### Summary

There was no effect of hand position on accuracy, sensitivity, bias, or response time. In general, the pattern of results was inconsistent with what was reported by Tseng and Bridgeman (2011). The null results of Experiment 3 do not appear to be the result of the grip posture and hand positions unique to holding a tablet and pushing touchscreen buttons.

## Discussion

Previous research has demonstrated that, in a variety of contexts, hand position can influence visual processing, including enhancing change detection, reducing the effect of distraction, and boosting sensitivity to low-spatial frequency information (e.g., Brockmole et al., 2013; Davoli & Brockmole, 2012; Reed et al., 2006; Tseng & Bridgeman, 2011). We aimed to explore the generalizability of this effect, specifically with respect to whether hand position could benefit the performance of a complex search task, and whether hand position effects might be observed when participants interacted with a tablet computer.

In two experiments, hand position was manipulated as participants searched for targets within x-ray images of baggage. In Experiment 1, on-screen response boxes required participants to hold the display between their hands in order to respond. In Experiment 2, participants were asked to sweep the display with their finger to encourage proximity between the target and their hand on each trial. Contrary to expectations, hand position did not result in any performance differences with respect to accuracy or target sensitivity. The instructed strategy in Experiment 2 resulted in longer inspection times but no improvement in target detection.

In Experiment 3, we attempted to replicate previously reported advantages in change detection performance when the hands were near the display. In three experiments, Tseng and Bridgeman (2011) observed a near-hands advantage with respect to sensitivity. A replication of their first experiment using a tablet computer and different grip postures and response inputs did not produce the same effect. Initially, we interpreted this as evidence that the specific device, grip postures, and responses used in our experiments may be responsible for the lack of a near-hands advantage. Previous experiments often had participants place their palms and fingers roughly parallel to the display, making a response by pushing their fingers/hands toward the center of the screen (e.g., Abrams et al., 2008; Tseng & Bridgeman, 2011). This might better simulate the grasping and reaching motions we typically make when interacting with objects in the world, compared to the responses participants made in our experiments in which they pushed their thumbs away from them to activate touchscreen buttons. As a result, our experiments may not have activated the neural systems required for enhanced visual processing. However, Experiment 4 tested this hypothesis with a direct replication of Tseng and Bridgeman (2011) in which participants responded to stimuli presented on a CRT monitor using buttons attached to the side of the display. Still, no near-hands advantage was observed.

## Conclusion

In general, our results suggest that the near-hands advantage may be very sensitive to small differences in procedure, which may have important implications for harnessing the near-hands advantage to produce better performance outside of the laboratory. Future research will be necessary to isolate the factors related to the reported null effects here. However, these four studies appear to demonstrate that, if such effects are to be harnessed to address applied problems, hand-position interventions need to be carefully considered. Hand proximity alone will not produce benefits even for relatively sparse and simple visual displays; benefits may instead depend on other factors.
